# Supplementary material for: Cohort-resolved excess mortality in Germany (2000-2024): Patterns and implications for the SARS-CoV-2 era
Source: PLoS One. 2025 Oct 27;20(10):e0334884. doi: 10.1371/journal.pone.0334884 (PMC12558607; doi:10.1371/journal.pone.0334884)
Supplement: S2 Fig — Females: German expected normalised AMRs (exNAMRs) with 5-year resolution of age cohorts; an exNAMR course is plotted over the years from 2000 through 2024 for a given CW (one line); see again the caption of Fig 1) for details on normalisation, fitting, and extrapolation of these courses. Fig A.2. Males: German expected normalised AMRs (exNAMRs) with 5-year resolution of age cohorts; an exNAMR course is plotted over the years from 2000 through 2024 for a given CW (one line); see again the caption of Fig 1) for details on normalisation, fitting, and extrapolation of these courses. Fig B.3. Females: German weekly observed AMCs (magenta lines), and correspondingly expected values (exAMCs, black lines) from 2000 through 2024, for the same seven age cohorts as in Figs 2, 3, 6. Fig B.4. Males: German weekly observed AMCs (magenta lines), and correspondingly expected values (exAMCs, black lines) from 2000 through 2024, for the same seven age cohorts as in Figs 2, 3, 6. Fig C.5. Females: German weekly NEAMRs (black lines) from 2000 through 2024, for the same seven age cohorts as in Figs 2, 3, 6; values exceeding (red) or dropping below (green) the 95% CI indicated by a spot. Fig C.6. Males: German weekly NEAMRs (black lines) from 2000 through 2024, for the same seven age cohorts as in Figs 2, 3, 6; values exceeding (red) or dropping below (green) the 95% CI indicated by a spot. Fig D.7. Females: German seasonal NEAMRs from 2000 through 2024, with 5-year resolution of age cohorts; NEAMR values calculated within three seasons constituting a year (top): ‘flu season 1’ (fls1: CW04-CW20), ‘summer season’ (sus: CW21-CW39), and ‘flu season 2’ (fls2: CW40-subsCW03); NEAMR values exceeding (reddish) or dropping below (greenish) the 95% CI (determined for each season) indicated by an asterisk or circle, respectively; bottom: the corresponding histograms for the time spans 2000-2019 (grey) and 2020-2024 (magenta), respectively, their arithmetic mean values symbolised by solid vertical [file pone.0334884.s002.pdf]

Supplementary Material S2

of

“Cohort-resolved excess mortality in Germany (2000-2024): Patterns and implications for the SARS-CoV-2 era”

by

Robert Rockenfeller and Michael Günther

A For both sexes separately, the age cohorts' normalised AMRs (NAMRs)

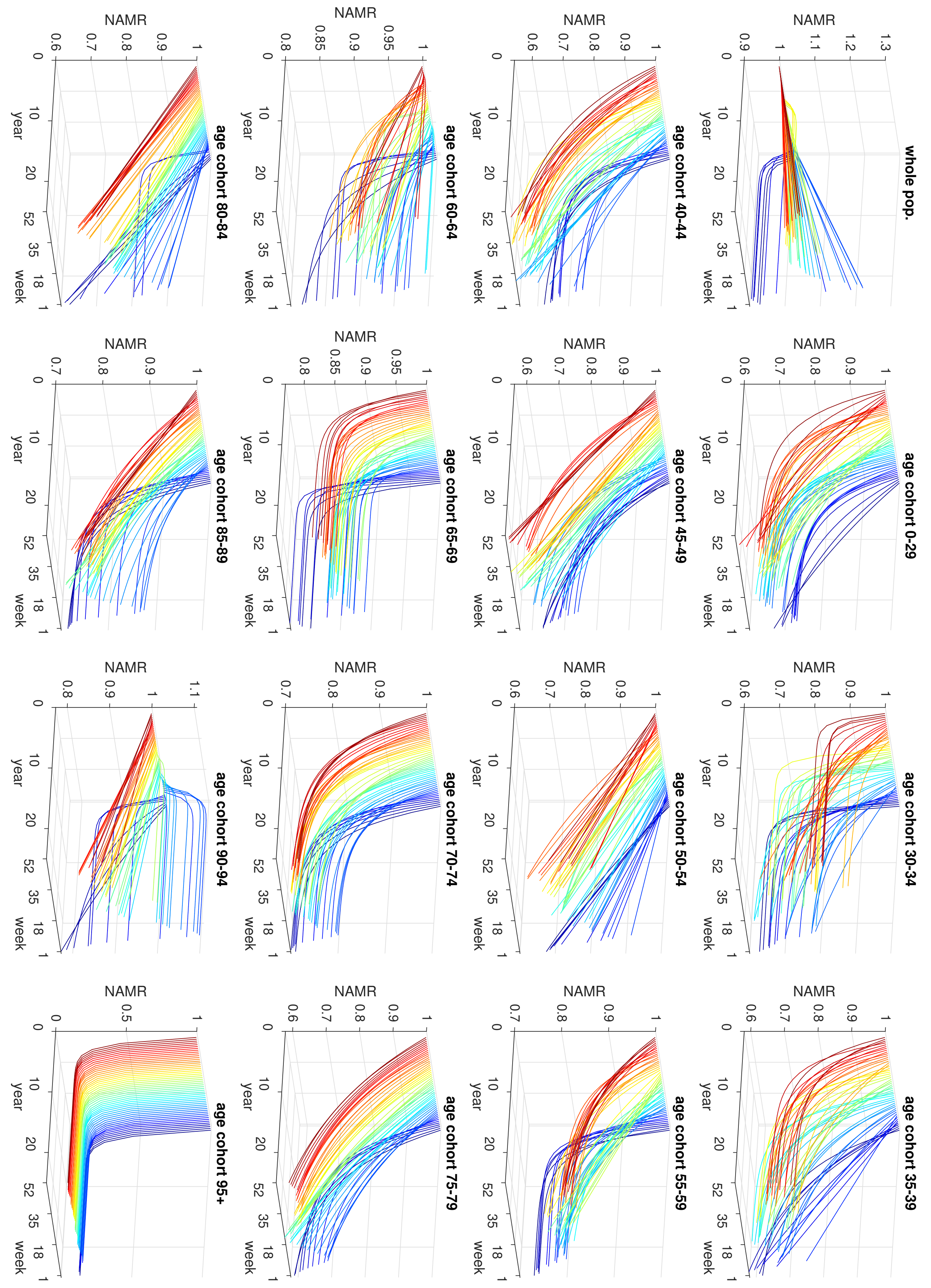

Figure A.1: *Females*: German expected normalised AMRs (exNAMRs) with 5-year resolution of age cohorts; an exNAMR course is plotted over the years from 2000 through 2024 for a given CW (one line); see again the caption of Fig. 1) for details on normalisation, fitting, and extrapolation of these courses.

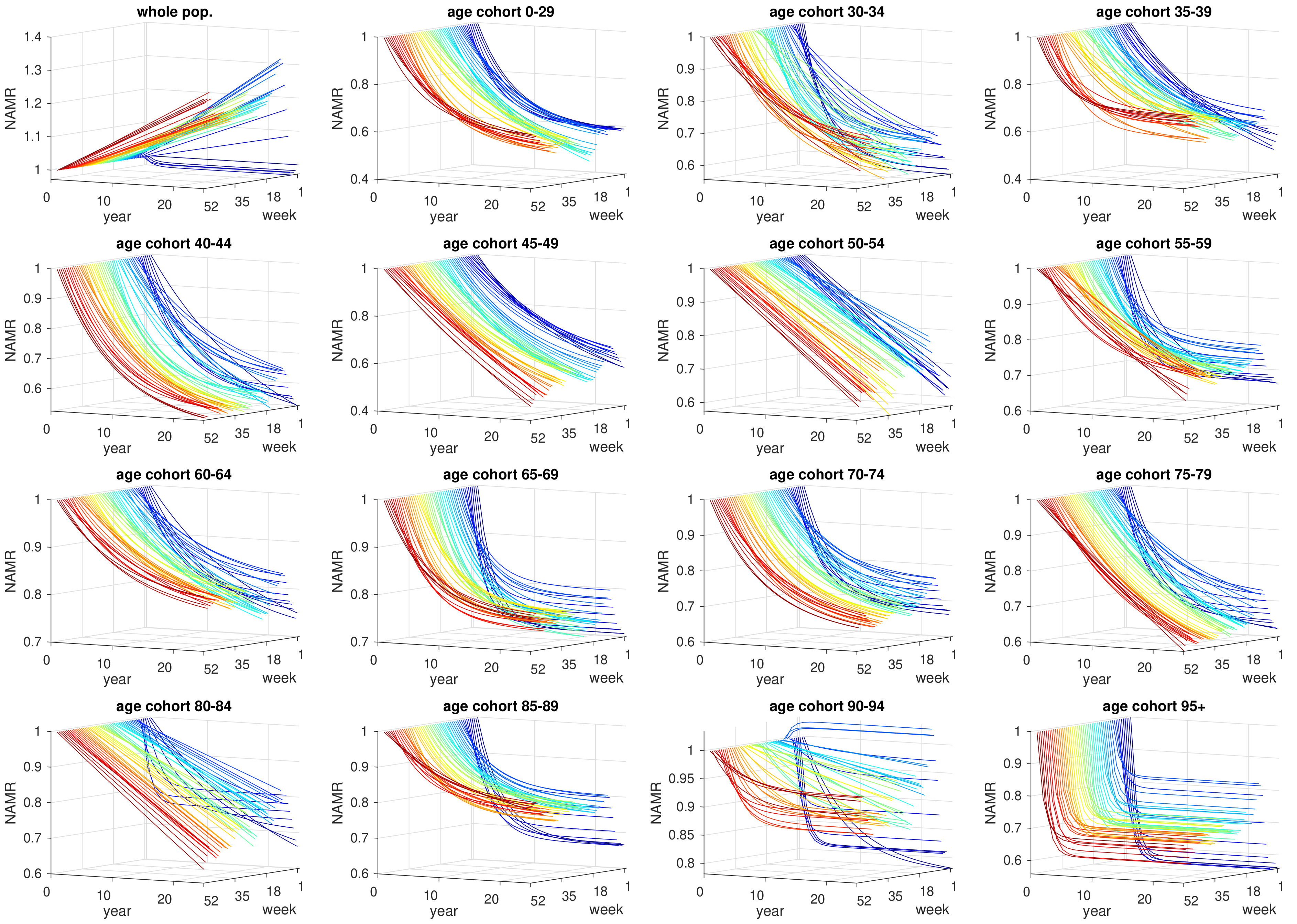

Figure A.2: *Males*: German expected normalised AMRs (exNAMRs) with 5-year resolution of age cohorts; an exNAMR course is plotted over the years from 2000 through 2024 for a given CW (one line); see again the caption of Fig. 1) for details on normalisation, fitting, and extrapolation of these courses.

B For both sexes separately, the age cohorts' weekly observed AMCs, and the correspondingly expected (model-estimated) values (exAMCs), from 2000 through 2024

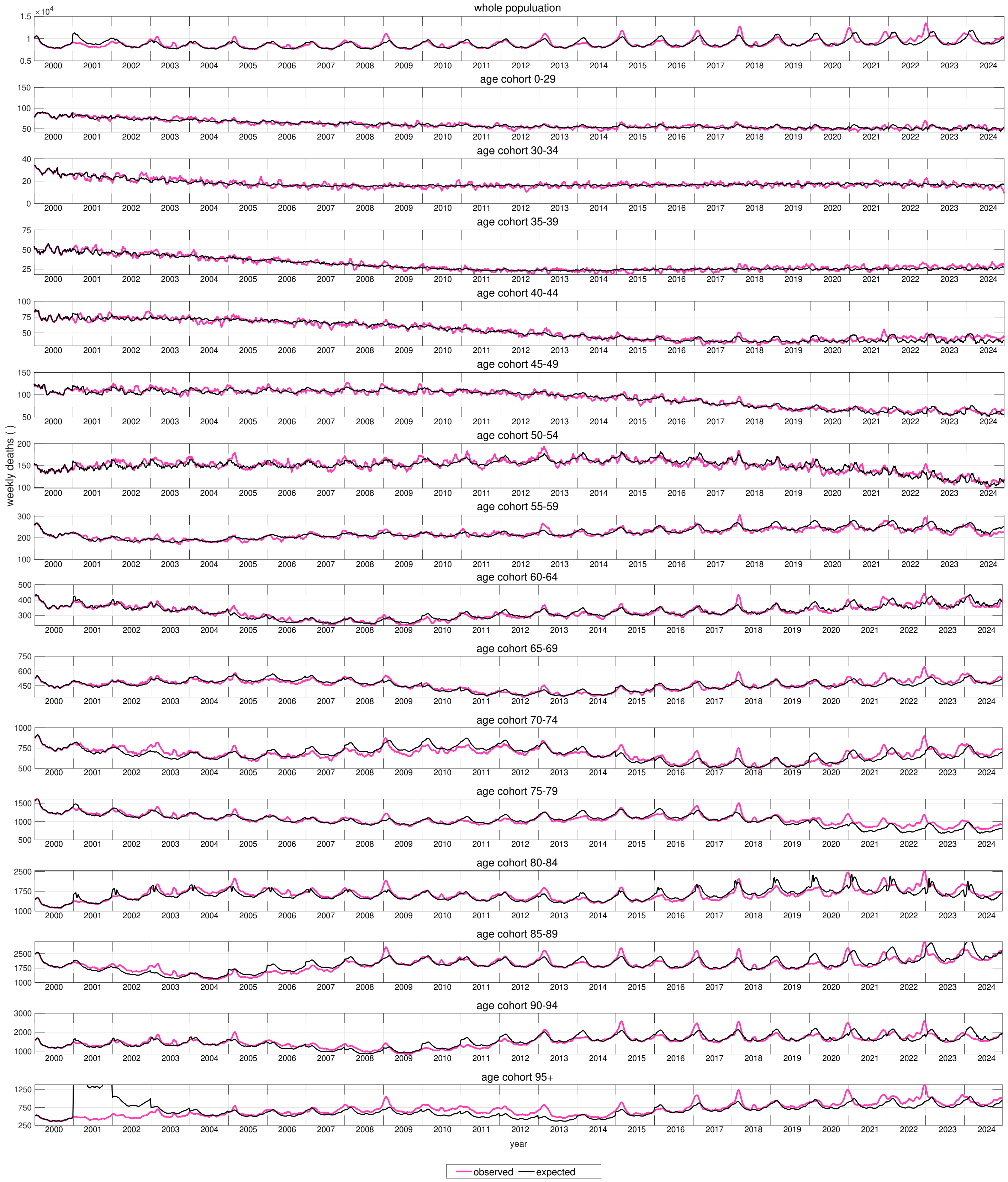

Figure B.3: *Females*: German weekly observed AMCs (magenta lines), and correspondingly expected values (exAMCs, black lines) from 2000 through 2024, for the same seven age cohorts as in Figs. 2,3,6.

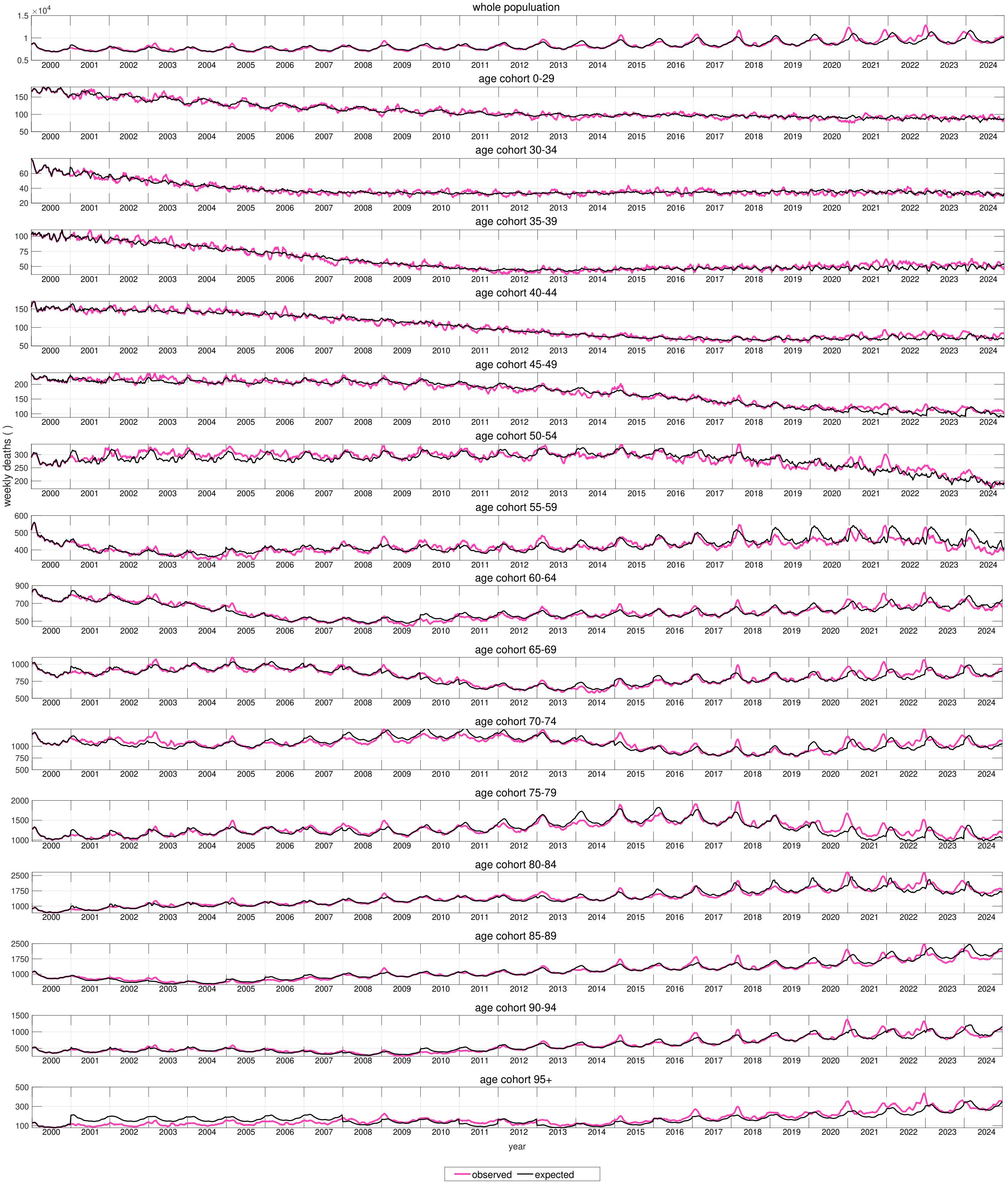

Figure B.4: *Males*: German weekly observed AMCs (magenta lines), and correspondingly expected values (exAMCs, black lines) from 2000 through 2024, for the same seven age cohorts as in Figs. 2,3,6.

C For both sexes separately, the age cohorts' weekly NEAMRs, from 2000 through 2024

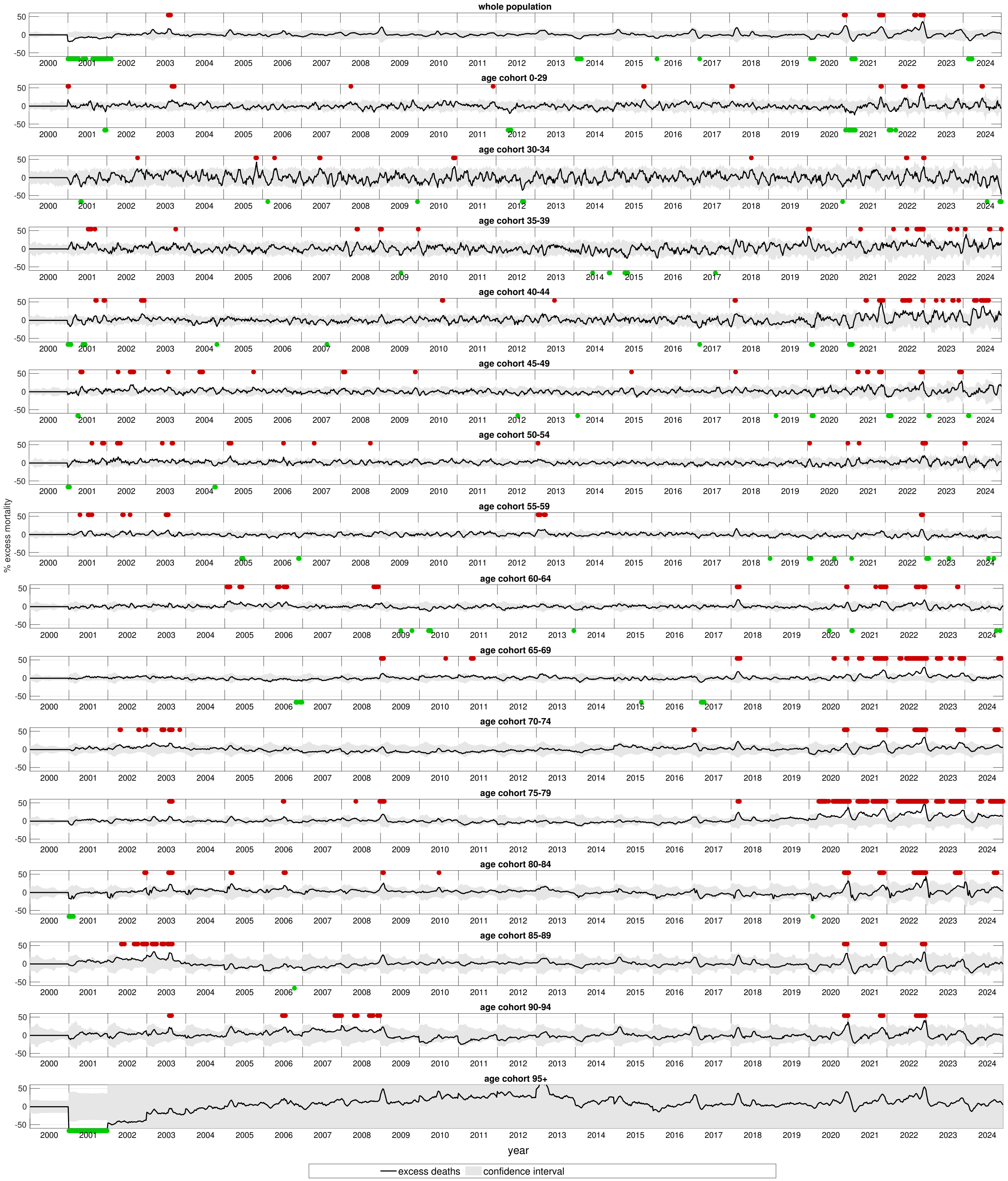

Figure C.5: *Females*: German weekly NEAMRs (black lines) from 2000 through 2024, for the same seven age cohorts as in Figs. 2,3,6; values exceeding (red) or dropping below (green) the 95% CI indicated by a spot.

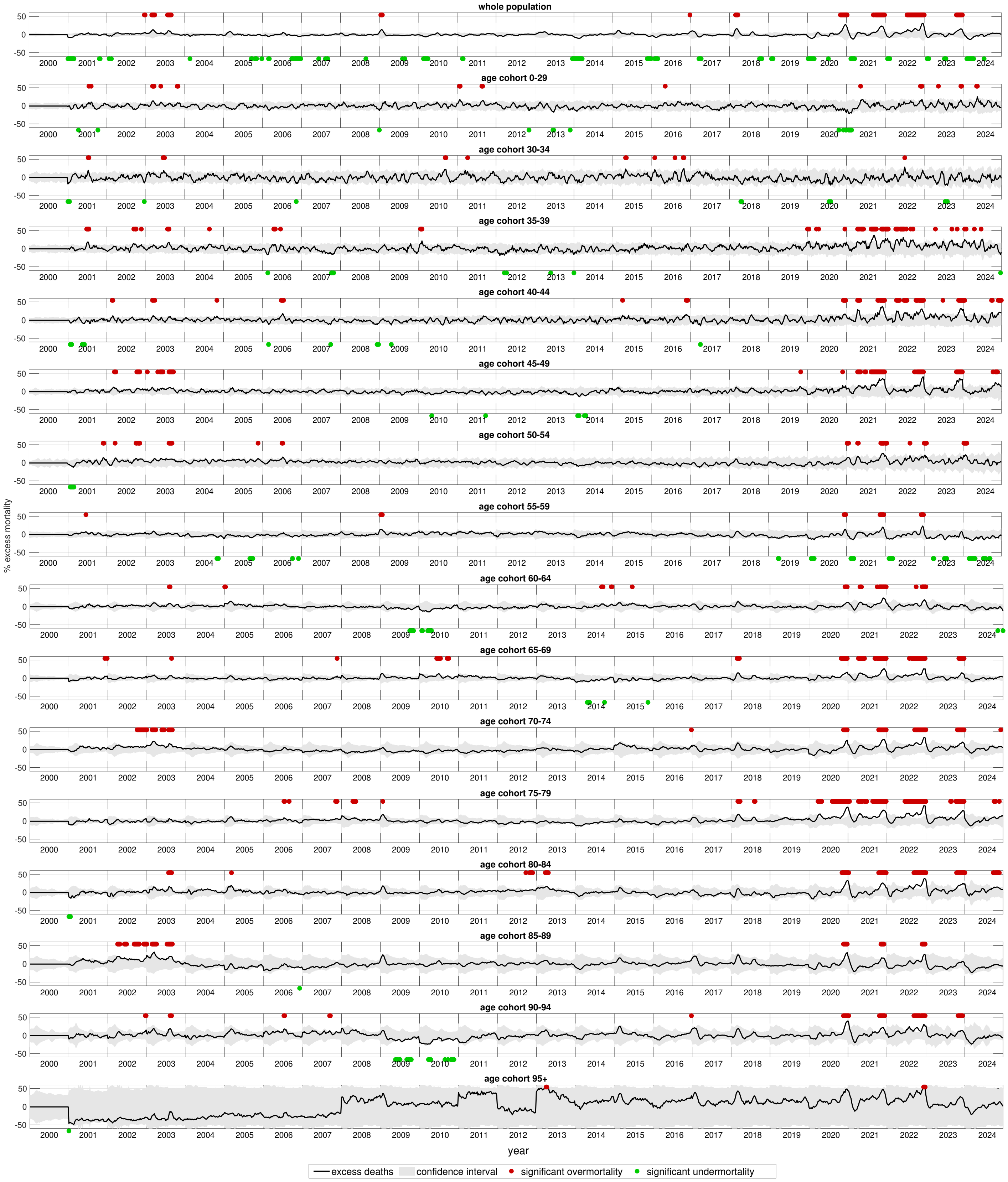

Figure C.6: *Males*: German weekly NEAMRs (black lines) from 2000 through 2024, for the same seven age cohorts as in Figs. 2,3,6; values exceeding (red) or dropping below (green) the 95% CI indicated by a spot.

D For both sexes separately, the age cohorts' seasonal NEAMRs, from 2000 through 2024

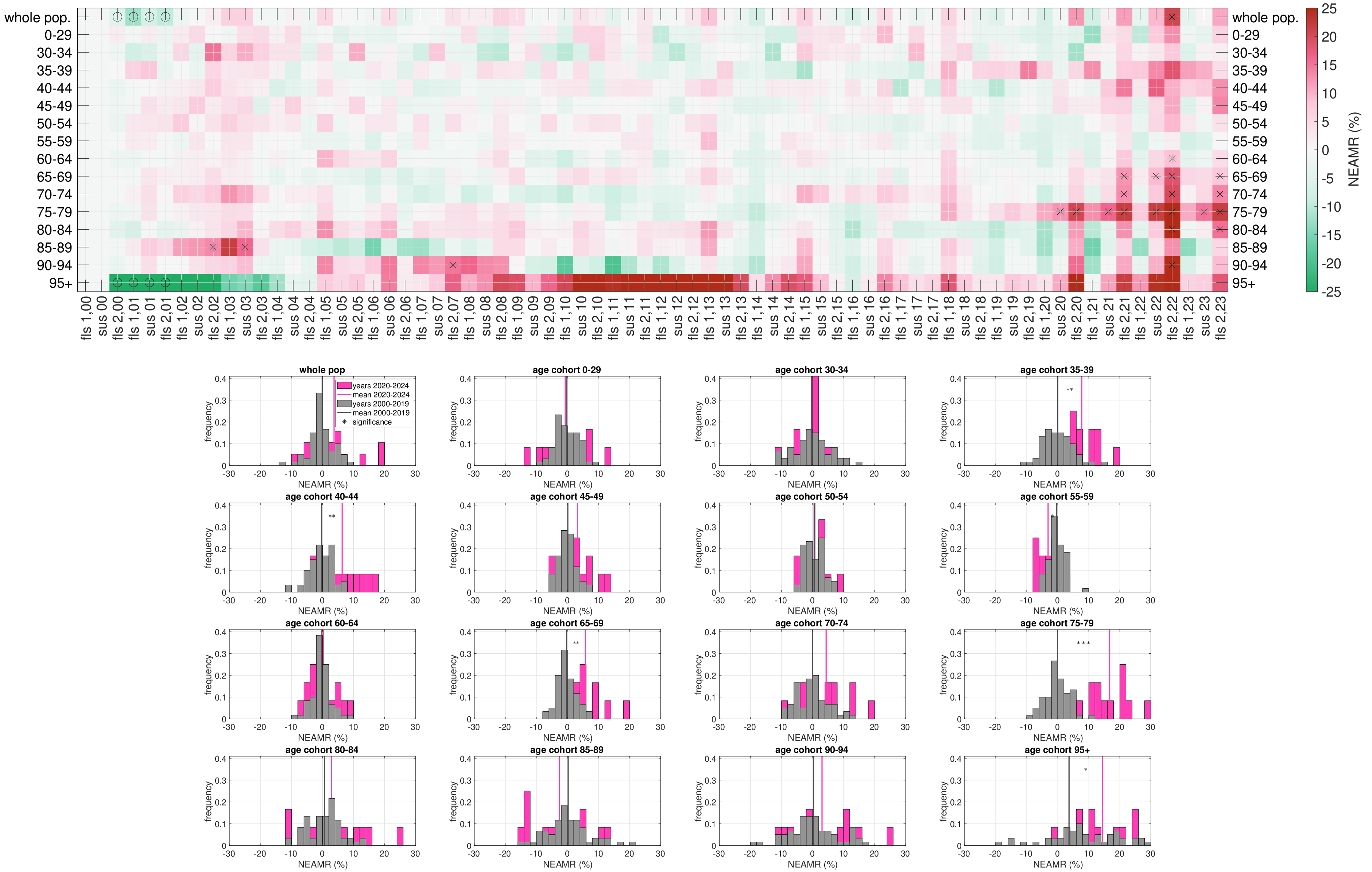

Figure D.7: *Females*: German seasonal NEAMRs from 2000 through 2024, with 5-year resolution of age cohorts; NEAMR values calculated within three seasons constituting a year (**top**): 'flu season 1' (fls_1: CW04-CW20), 'summer season' (sus: CW21-CW39), and 'flu season 2' (fls_2: CW40-subsCW03); NEAMR values exceeding (reddish) or dropping below (greenish) the 95% CI (determined for each season) indicated by an asterisk or circle, respectively; **bottom**: the corresponding histograms for the time spans 2000-2019 (grey) and 2020-2024 (magenta), respectively, their arithmetic mean values symbolised by solid vertical lines; the significance of the difference in mean values is indicated by star symbols: $p < 0.05$ (one), $p < 0.01$ (two), $p < 0.001$ (three).

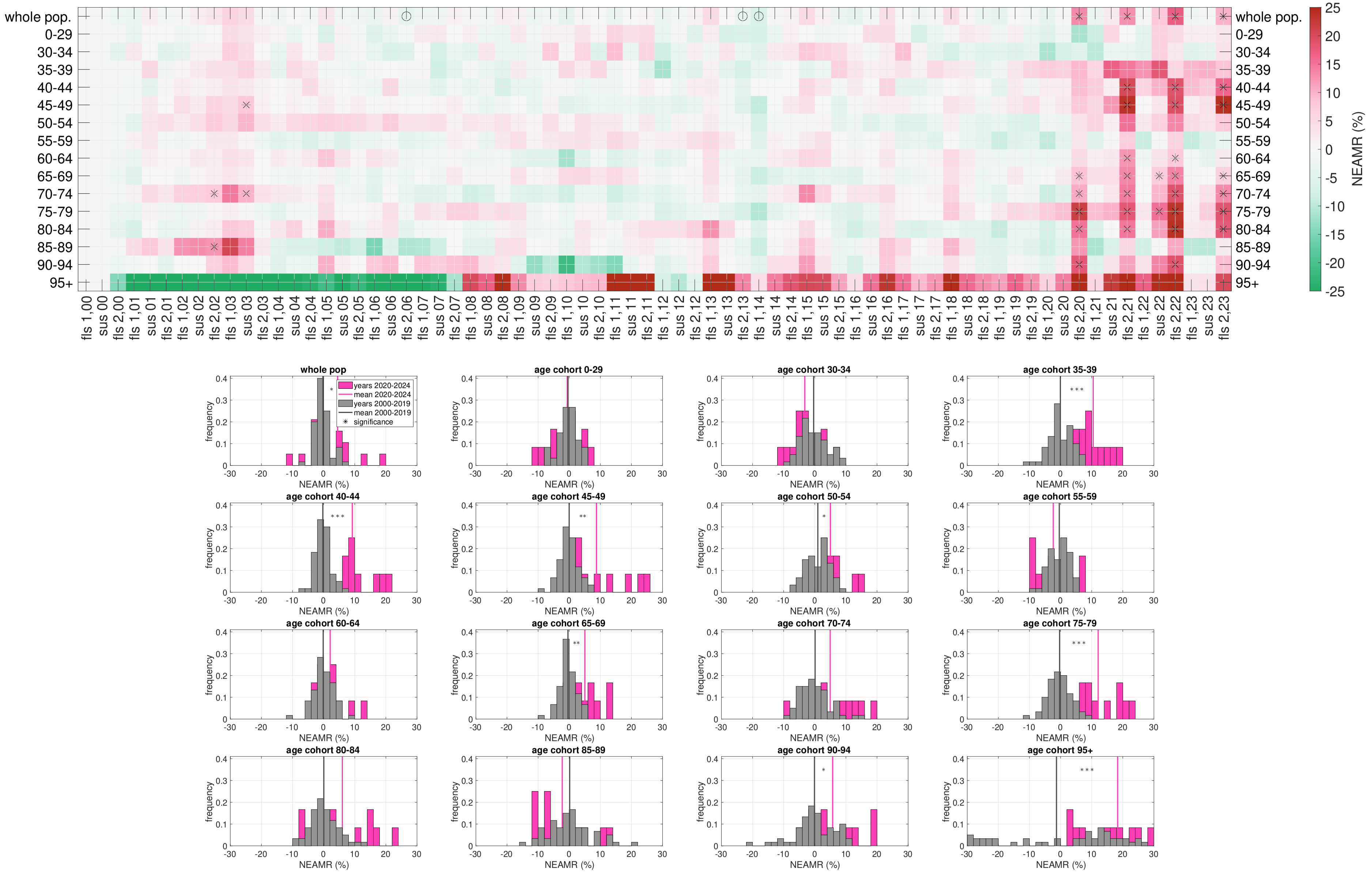

Figure D.8: *Males*: German seasonal NEAMRs from 2000 through 2024, with 5-year resolution of age cohorts; NEAMR values calculated within three seasons constituting a year (**top**): 'flu season 1' (fls_1: CW04-CW20), 'summer season' (sus: CW21-CW39), and 'flu season 2' (fls_2: CW40-subsCW03); NEAMR values exceeding (reddish) or dropping below (greenish) the 95% CI (determined for each season) indicated by an asterisk or circle, respectively; **bottom**: the corresponding histograms for the time spans 2000-2019 (grey) and 2020-2024 (magenta), respectively, their arithmetic mean values symbolised by solid vertical lines; the significance of the difference in mean values is indicated by star symbols: $p < 0.05$ (one), $p < 0.01$ (two), $p < 0.001$ (three).

E For both sexes separately, the age cohorts' weekly NEAMRs, from 2020 through 2024, as well as incidences of (SARS-CoV-2-variant-specific) PCR+ and mRNA-I

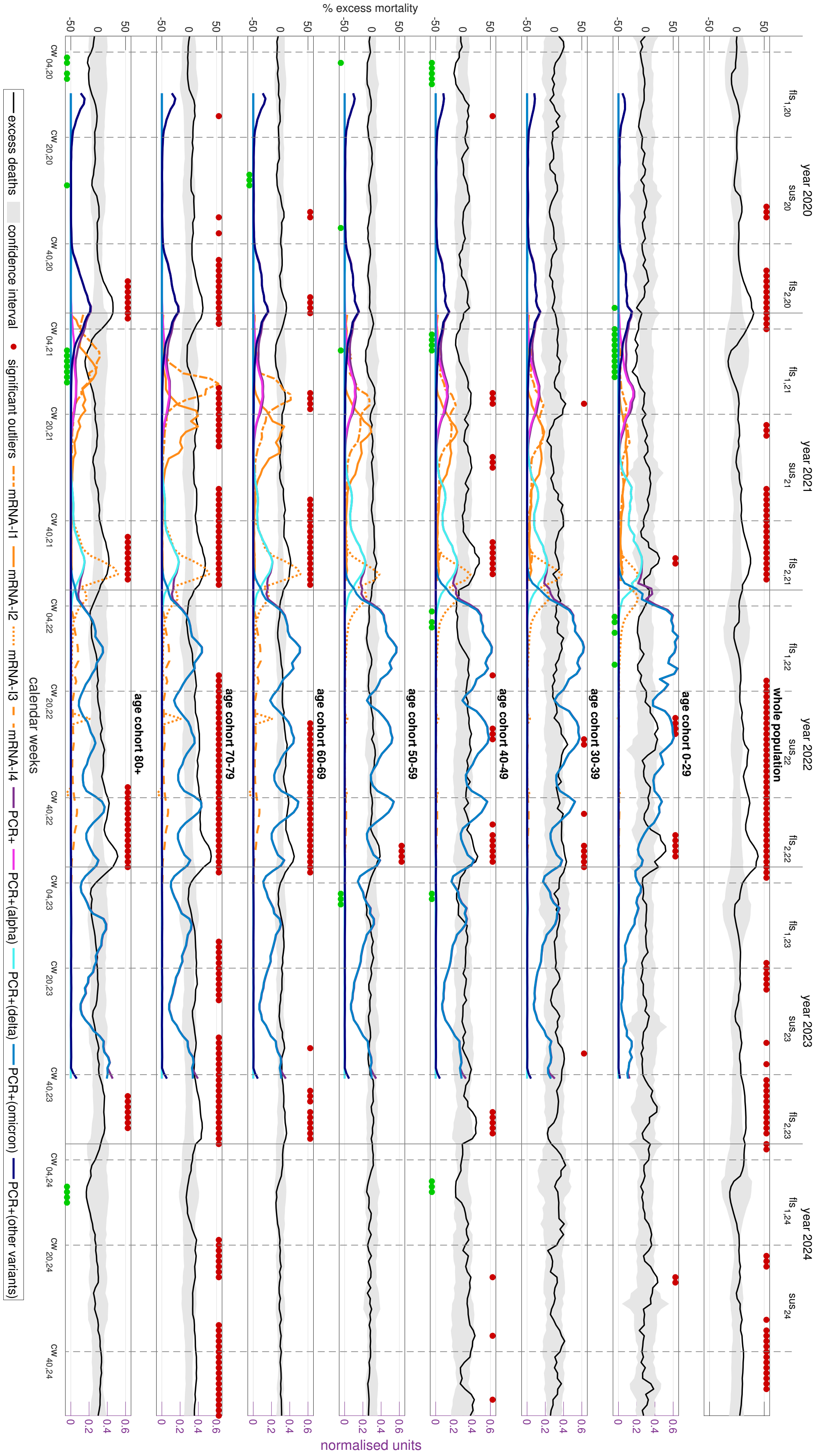

Figure E.9: *Females*: German weekly NEAMRs (black lines) from 2020 through 2024, for the same seven age cohorts as in Figs. 2,3,6; see caption of Fig. 6 for detailed explanation of all symbols shown.

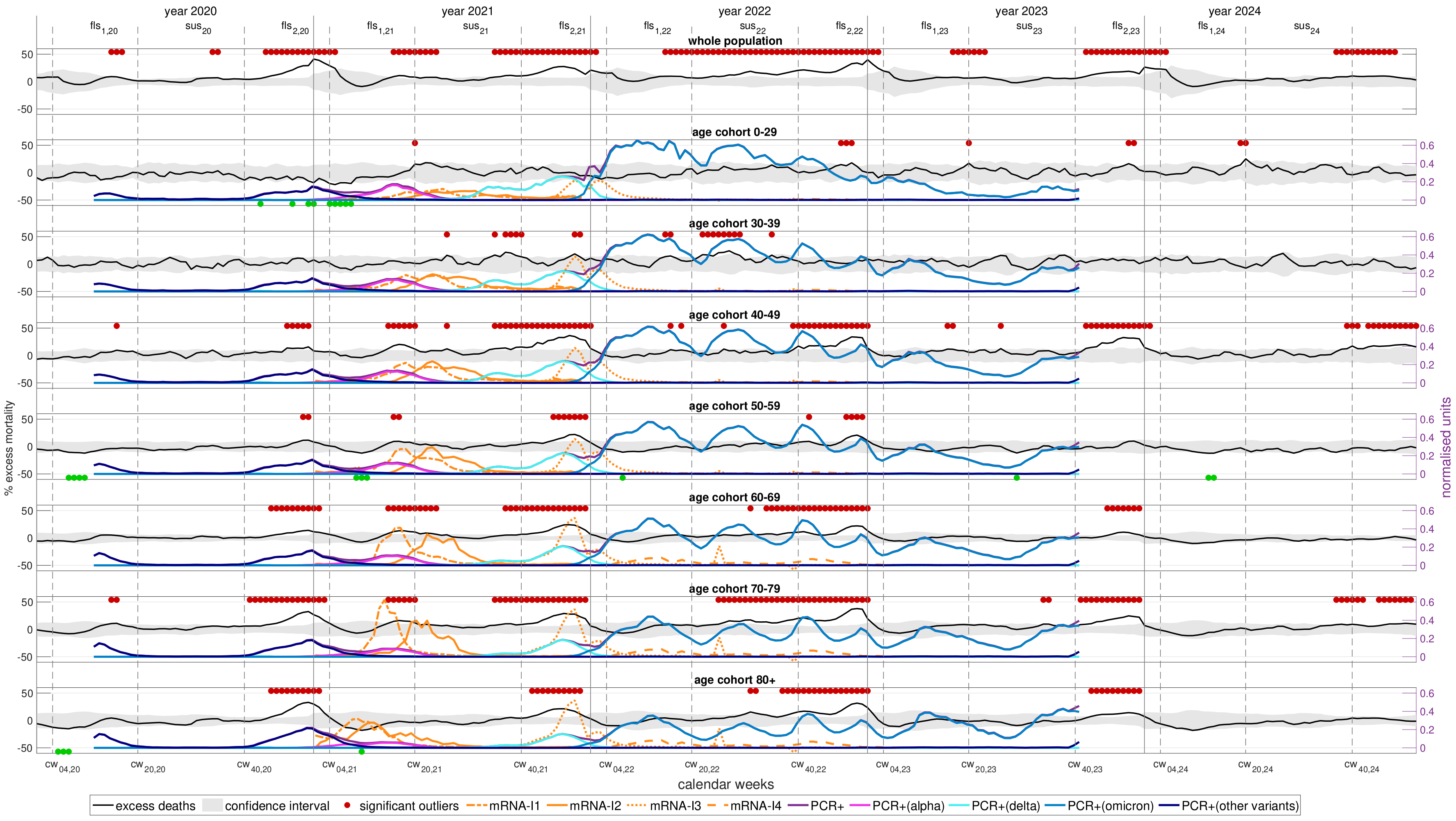

Figure E.10: *Males*: German weekly NEAMRs (black lines) from 2020 through 2024, for the same seven age cohorts as in Figs. 2,3,6; see caption of Fig. 6 for detailed explanation of all symbols shown.

F For both sexes separately, cross-correlations during CW04-CW42,2021 of the age cohorts' NEAMR and some incidence signals

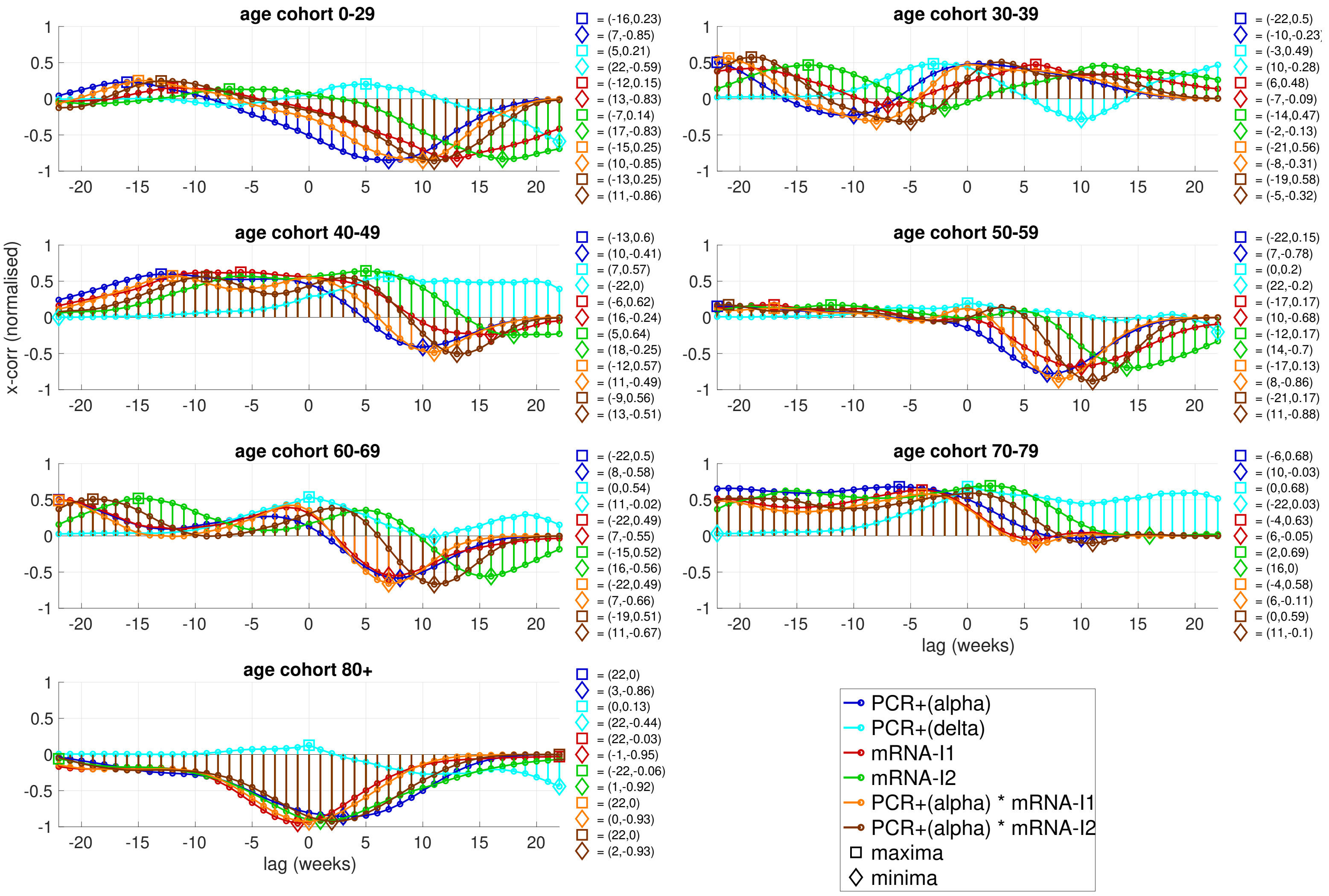

Figure F.11: In Germany, during the time interval CW04-CW42,2021, and for seven *female* age cohorts, the (Pearson) coefficients of cross-correlating their respective weekly NEAMR time course with several (time-lagged) normalised incidence signals (regarding normalisation, see caption of Fig. 6) are plotted: with the *normalised weekly numbers of positive PCR tests* PCR+(V) of two SARS-CoV-2 variants V=alpha,delta, and with the *normalised weekly number of mRNA-I* of the first (mRNA-I1), and second (mRNA-I2) mRNA-I, as well as with two products by week of incidences; maximum and minimum coefficient values to the right of a sub-panel; the same for both sexes together: see again Fig. 7.

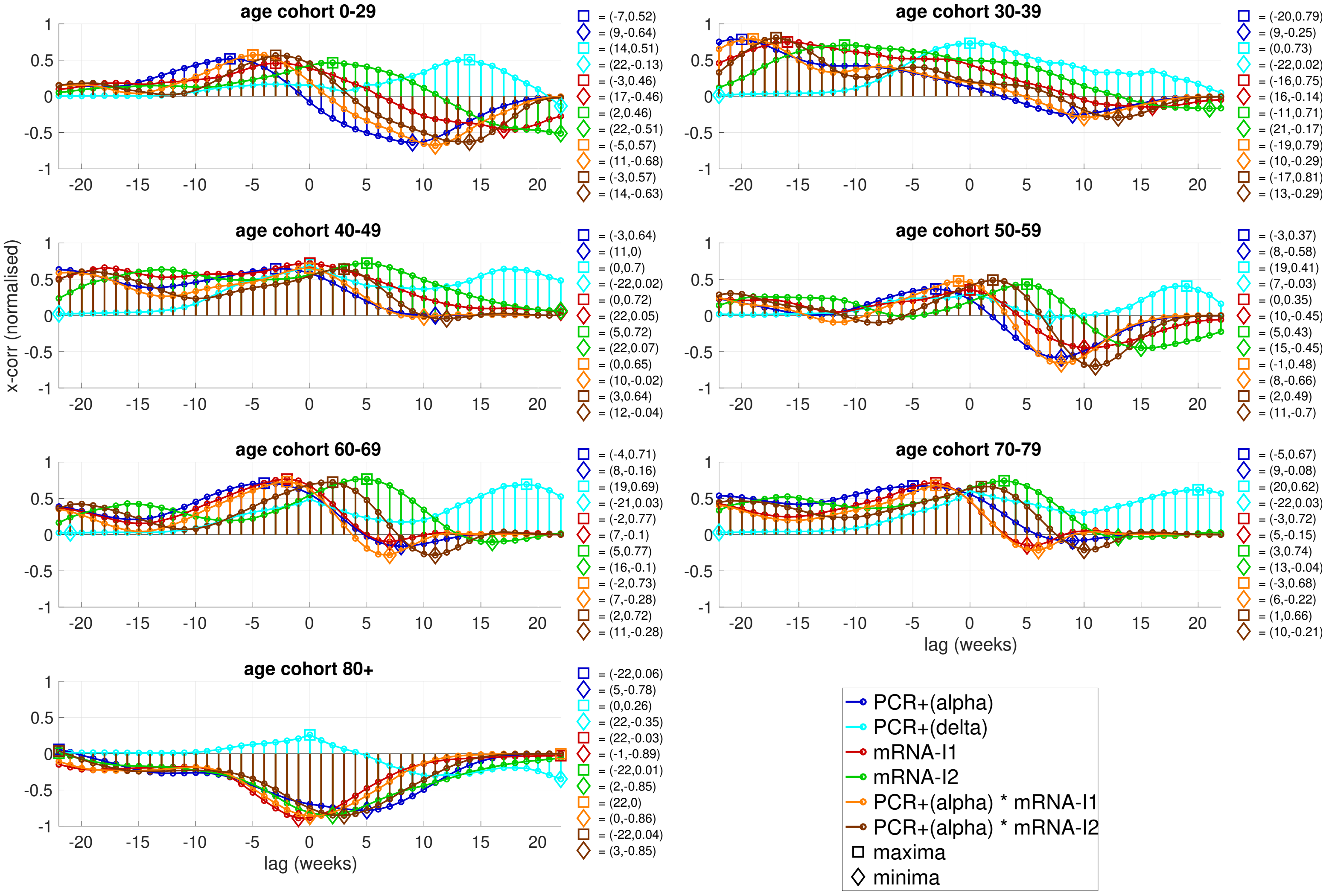

Figure F.12: In Germany, during the time interval CW04-CW42,2021, and for seven *male* age cohorts, the (Pearson) coefficients of cross-correlating their respective weekly NEAMR time course with several (time-lagged) normalised incidence signals (regarding normalisation, see caption of Fig. 6) are plotted: with the *normalised weekly numbers of positive PCR tests* PCR+(V) of two SARS-CoV-2 variants V=alpha,delta, and with the *normalised weekly number of mRNA-I* of the first (mRNA-I1), and second (mRNA-I2) mRNA-I, as well as with two products by week of incidences; maximum and minimum coefficient values to the right of a sub-panel; the same for both sexes together: see again Fig. 7.

G For both sexes separately, cross-correlations during CW30,2021-CW03,2022 of the age cohorts' NEAMR and some incidence signals

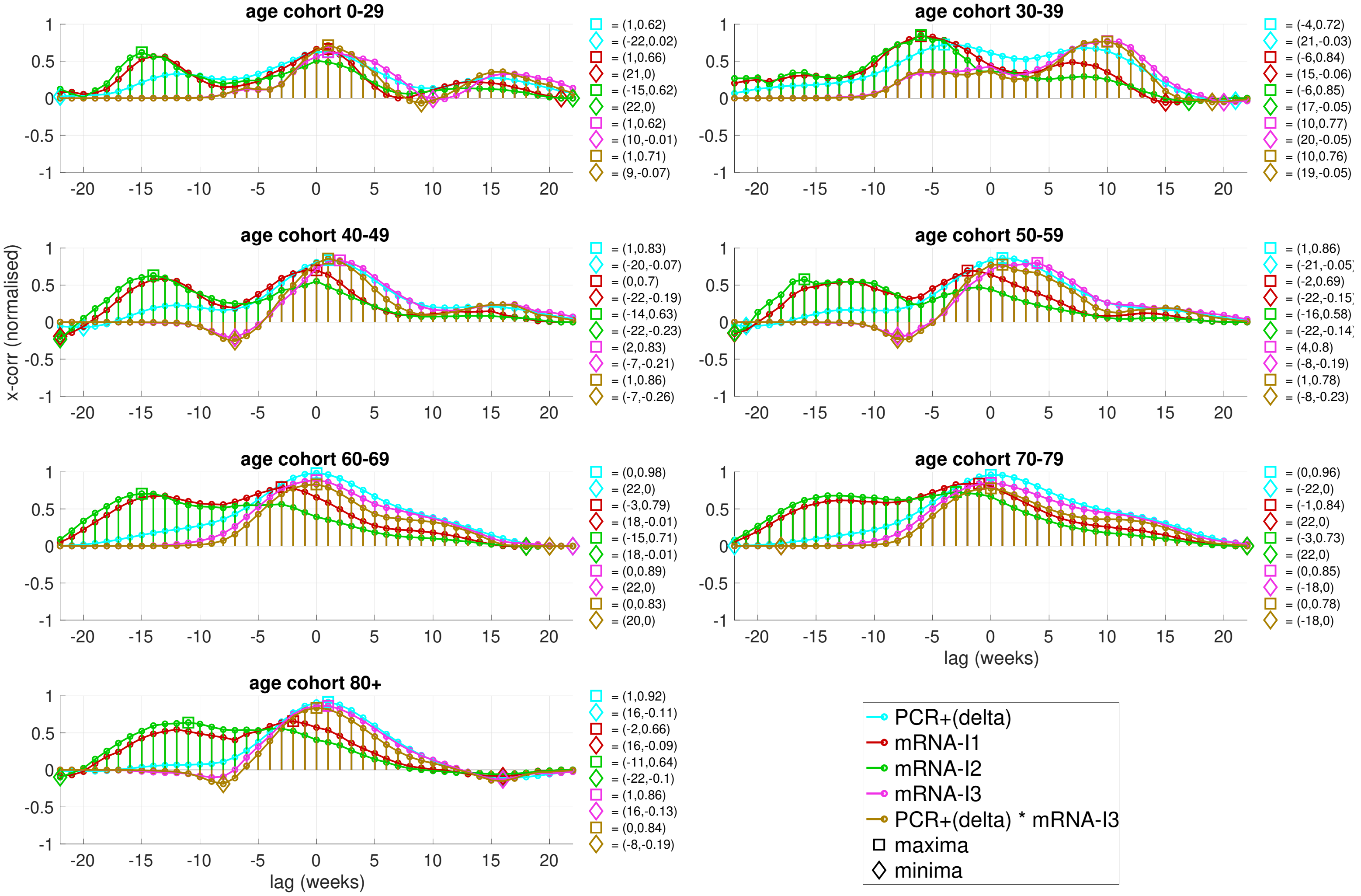

Figure G.13: In Germany, during the time interval CW30,2021-CW03,2022, and for seven *female* age cohorts, the (Pearson) coefficients of cross-correlating their respective weekly NEAMR time course with several (time-lagged) normalised incidence signals (regarding normalisation, see caption of Fig. 6) are plotted: with the *normalised weekly numbers of positive PCR tests* PCR+(V) of the SARS-CoV-2 variant V=delta, and with the *normalised weekly number of mRNA-I* of the first (mRNA-I1), second (mRNA-I2), and third (mRNA-I3) mRNA-I, as well as with one product by week of incidences; maximum and minimum coefficient values to the right of a sub-panel; the same for both sexes together: see again Fig. 8.

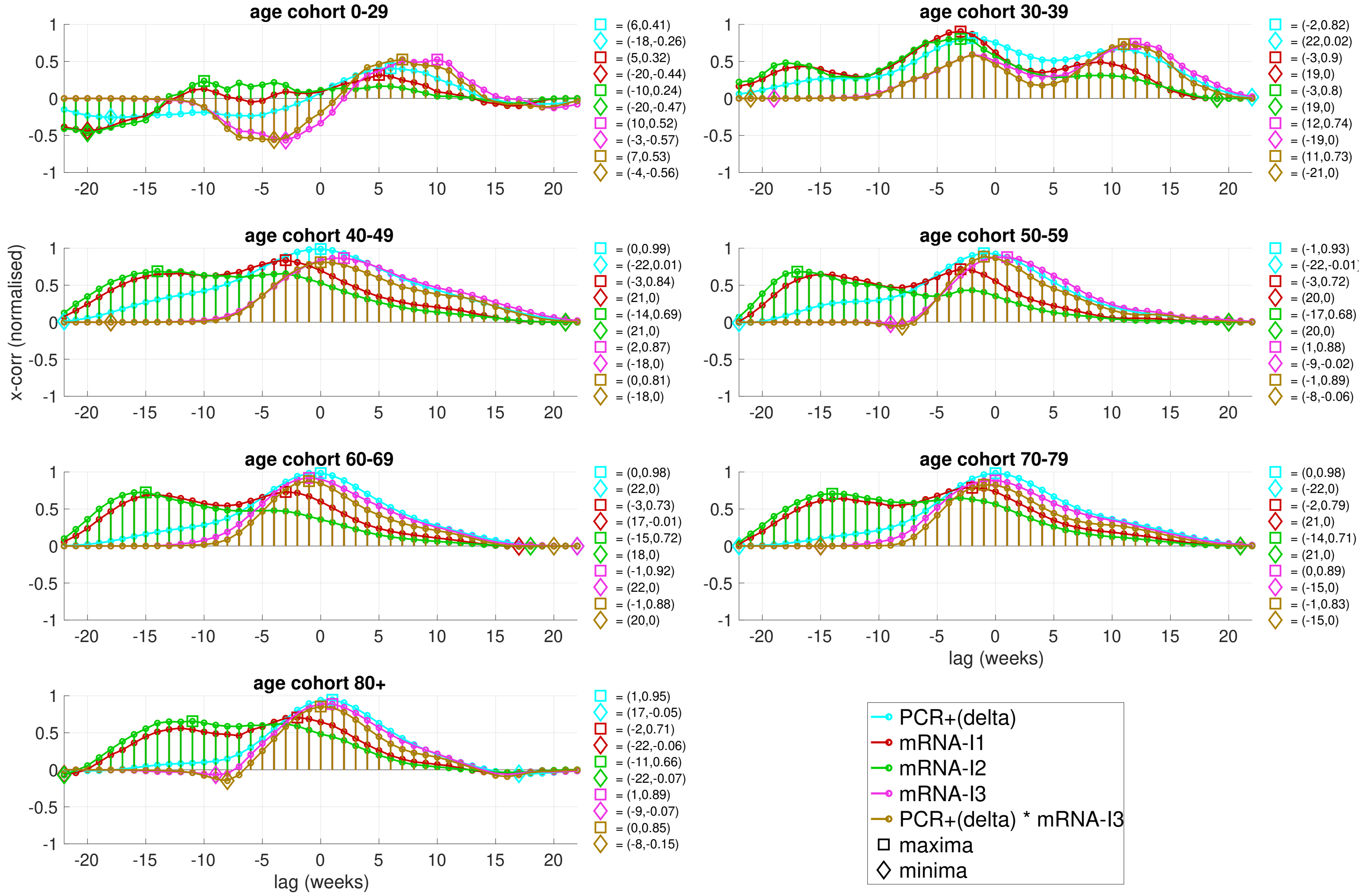

Figure G.14: In Germany, during the time interval CW30,2021-CW03,2022, and for seven *male* age cohorts, the (Pearson) coefficients of cross-correlating their respective weekly NEAMR time course with several (time-lagged) normalised incidence signals (regarding normalisation, see caption of Fig. 6) are plotted: with the *normalised weekly numbers of positive PCR tests* PCR+(V) of the SARS-CoV-2 variant V=delta, and with the *normalised weekly number of mRNA-I* of the first (mRNA-I1), second (mRNA-I2), and third (mRNA-I3) mRNA-I, as well as with one product by week of incidences; maximum and minimum coefficient values to the right of a sub-panel; the same for both sexes together: see again Fig. 8.
